# 2-((*E*)-{(*S*)-(6-Meth­oxy­quinolin-4-yl)[(2*S*)-8-vinyl­quinuclidin-2-yl]methyl­imino}­meth­yl)phenol

**DOI:** 10.1107/S1600536811021507

**Published:** 2011-06-18

**Authors:** Yu Wei, Wei He

**Affiliations:** a Department of Chemistry, School of Pharmacy, Fourth Military Medical University, Shaanxi Province, Xi’an 710032, People’s Republic of China

## Abstract

The title compound, C_27_H_29_N_3_O_2_, adopts an *E* configuration with respect to the C=N bond. The molecular structure is stabilized by inter­molecular O—H⋯N inter­actions between a hy­droxy H atom and the N atom on the quinoline ring.

## Related literature

For literature on the preparation of Schiff base compounds, see: Jennings & Lovely (1991 ▶); Yoon & Jacobsen (2005 ▶). For the uses of Schiff base compounds, see: Yin *et al.* (2004 ▶). For the crystal structures of Schiff base compounds, see: Zhu (2011 ▶); Xie *et al.* (2010 ▶). For reference bond values, see: Jones (1986 ▶); Hooft *et al.* (2008 ▶). For information on the absolute structure of the title compound, see: Brunner *et al*. (1995 ▶); He *et al*. (2006 ▶).
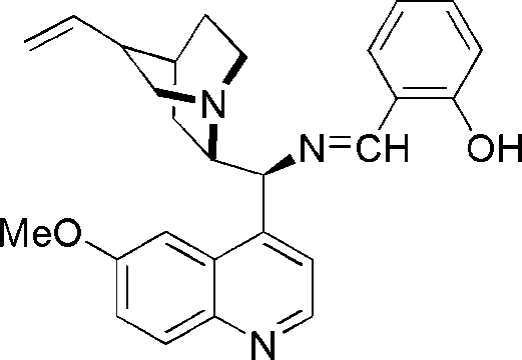


## Experimental

### 

#### Crystal data

C_27_H_29_N_3_O_2_
*M* *_r_* = 427.53
Orthorhombic, 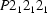

*a* = 8.9285 (15) Å
*b* = 11.6759 (19) Å
*c* = 21.939 (4) Å
*V* = 2287.1 (7) Å^3^
*Z* = 4
Mo *K*α radiation
μ = 0.08 mm^−1^
*T* = 296 K
0.35 × 0.29 × 0.17 mm

#### Data collection

Bruker APEXII CCD diffractometer
Absorption correction: multi-scan (*SADABS*; Bruker, 2005 ▶) *T*_min_ = 0.973, *T*_max_ = 0.987
11474 measured reflections
2339 independent reflections
2098 reflections with *I* > 2σ(*I*)
*R*_int_ = 0.026

#### Refinement

*R*[*F* ^2^ > 2σ(*F* ^2^)] = 0.032
*wR*(*F* ^2^) = 0.084
*S* = 1.05
2339 reflections
292 parameters
H-atom parameters constrained
Δρ_max_ = 0.10 e Å^−3^
Δρ_min_ = −0.10 e Å^−3^

### 

Data collection: *APEX2* (Bruker, 2008 ▶); cell refinement: *SAINT* (Bruker, 2008 ▶); data reduction: *SAINT*; program(s) used to solve structure: *SHELXS97* (Sheldrick, 2008 ▶); program(s) used to refine structure: *SHELXL97* (Sheldrick, 2008 ▶); molecular graphics: *SHELXTL* (Sheldrick, 2008 ▶); software used to prepare material for publication: *Mercury* (Macrae *et al.*, 2006 ▶).

## Supplementary Material

Crystal structure: contains datablock(s) I, global. DOI: 10.1107/S1600536811021507/jh2284sup1.cif

Structure factors: contains datablock(s) I. DOI: 10.1107/S1600536811021507/jh2284Isup2.hkl

Supplementary material file. DOI: 10.1107/S1600536811021507/jh2284Isup3.cml

Additional supplementary materials:  crystallographic information; 3D view; checkCIF report

## Figures and Tables

**Table 1:** Hydrogen-bond geometry (Å, °)

*D*—H⋯*A*	*D*—H	H⋯*A*	*D*⋯*A*	*D*—H⋯*A*
O1—H1⋯N1	0.82	1.88	2.605 (2)	148

**Fig. 1.**
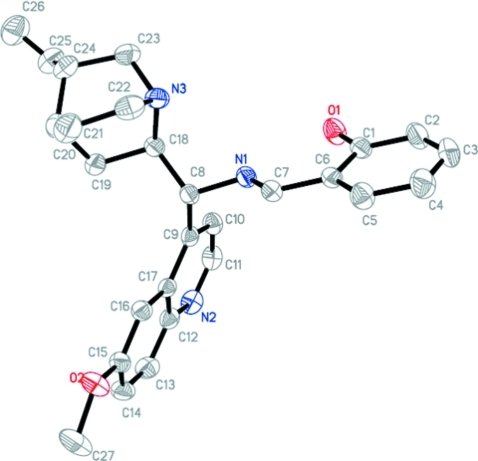
The molecular structure of the title compound.

## supplementary crystallographic information

### Comment

In recent years, considerable attention has been focused on the Schiff-base ligands, *e.g.* as organocatalysts or ligands of metal complexes in asymmetric reactions; as biological active compounds owing to their anti-tumour abilities (Yin *et al.*, 2004). We report here the crystal structure of the title Schiff-base compound (Fig. 1).

The molecule of the compound adopts an *E* configuration with respect to the C═N bond. The dihedral angle between the quinoline ring and the part of spirane C8C18C24 is 63.06°. The dihedral angle between benzene ring and quinoline ring is 65.20°. And it is 54.46° between benzene ring and the spirane part C8C18C24. All the bond lengths are within normal values (Jones, 1986; Hooft *et al.*, 2008), and are comparable with those in the similar *Cinchona* alkaloid-derived Schiff base compounds as cited above (Zhu, 2011; Xie *et al.*, 2010). The molecular conformation is stabilized by O—H···N interactions (Table 1).

### Experimental

Salicylaldehyde (0.24 ml, 2.3 mmol) and 9-amino-(9-deoxy)-epiquinine (0.513 g, 1.588 mmol) in toluene (40 ml) was heated to reflux. After that, two scoops of Al_2_O_3_ (about 1.5 g, dried at 110 °C for two hours before use) were added to the solution. And then added one more scoop each hour. After four hours, the temperature was slowly cooling down to room temperature. Then the mixture was filtrated and the residue was washed with Et_2_O. The combined organic layers were removed under reduced pressure. The residue was purified by flash chromatography on silica gel (CH_2_Cl_2_/methanol/Et_3_N 30/1/1) to afford Schiff base compound 1 b (570 mg, 84% yield) as a yellow solid. HRMS (ESI, *M*+H) calcd for C_27_H_30_N_3_O_2_ 428.2338, found 428.2333.

### Refinement

All H atoms were placed in their calculated positions and then refined using the riding model approximation, with C—H lengths of 0.93Å (CH), 0.97Å (CH2), 0.96Å (CH3), and *U*iso(H) = 1.2*U*eq(C) or *U*iso(H) = 1.5*U*eq(C27).

### Crystal data

**Table:**

C_27_H_29_N_3_O_2_	*D*_x_ = 1.242 Mg m^−^^3^
*M**_r_* = 427.53	Melting point: 438(1) K
Orthorhombic, *P*2_1_2_1_2_1_	Mo *K*α radiation, λ = 0.71073 Å
*a* = 8.9285 (15) Å	Cell parameters from 4474 reflections
*b* = 11.6759 (19) Å	θ = 2.5–27.8°
*c* = 21.939 (4) Å	µ = 0.08 mm^−^^1^
*V* = 2287.1 (7) Å^3^	*T* = 296 K
*Z* = 4	Block, yellow
*F*(000) = 912	0.35 × 0.29 × 0.17 mm

### Data collection

**Table:**

Bruker APEXII CCD diffractometer	2339 independent reflections
Radiation source: fine-focus sealed tube	2098 reflections with *I* > 2σ(*I*)
graphite	*R*_int_ = 0.026
φ and ω scans	θ_max_ = 25.1°, θ_min_ = 1.9°
Absorption correction: multi-scan (*SADABS*; Bruker, 2005)	*h* = −9→10
*T*_min_ = 0.973, *T*_max_ = 0.987	*k* = −13→12
11474 measured reflections	*l* = −26→23

### Refinement

**Table:**

Refinement on *F*^2^	Secondary atom site location: difference Fourier map
Least-squares matrix: full	Hydrogen site location: inferred from neighbouring sites
*R*[*F*^2^ > 2σ(*F*^2^)] = 0.032	H-atom parameters constrained
*wR*(*F*^2^) = 0.084	*w* = 1/[σ^2^(*F*_o_^2^) + (0.044*P*)^2^ + 0.2035*P*] where *P* = (*F*_o_^2^ + 2*F*_c_^2^)/3
*S* = 1.05	(Δ/σ)_max_ < 0.001
2339 reflections	Δρ_max_ = 0.10 e Å^−^^3^
292 parameters	Δρ_min_ = −0.10 e Å^−^^3^
0 restraints	Extinction correction: *SHELXL97* (Sheldrick, 2008), Fc^*^=kFc[1+0.001xFc^2^λ^3^/sin(2θ)]^-1/4^
Primary atom site location: structure-invariant direct methods	Extinction coefficient: 0.0073 (11)

### Special details

**Table:**

Geometry. All e.s.d.'s (except the e.s.d. in the dihedral angle between two l.s. planes) are estimated using the full covariance matrix. The cell e.s.d.'s are taken into account individually in the estimation of e.s.d.'s in distances, angles and torsion angles; correlations between e.s.d.'s in cell parameters are only used when they are defined by crystal symmetry. An approximate (isotropic) treatment of cell e.s.d.'s is used for estimating e.s.d.'s involving l.s. planes.
Refinement. Refinement of *F*^2^ against ALL reflections. The weighted *R*-factor *wR* and goodness of fit *S* are based on *F*^2^, conventional *R*-factors *R* are based on *F*, with *F* set to zero for negative *F*^2^. The threshold expression of *F*^2^ > σ(*F*^2^) is used only for calculating *R*-factors(gt) *etc*. and is not relevant to the choice of reflections for refinement. *R*-factors based on *F*^2^ are statistically about twice as large as those based on *F*, and *R*- factors based on ALL data will be even larger.

### Fractional atomic coordinates and isotropic or equivalent isotropic displacement parameters (Å^2^)

**Table:**

	*x*	*y*	*z*	*U*_iso_*/*U*_eq_	
N1	0.41218 (19)	0.89212 (15)	0.85006 (7)	0.0446 (4)	
N2	0.2454 (2)	1.09111 (16)	0.66032 (9)	0.0596 (5)	
N3	0.7031 (2)	0.98276 (17)	0.88131 (7)	0.0534 (5)	
O1	0.2162 (2)	0.92618 (13)	0.93612 (7)	0.0639 (4)	
H1	0.2749	0.9431	0.9088	0.096*	
O2	0.6132 (2)	0.73226 (15)	0.59318 (7)	0.0676 (5)	
C1	0.2036 (2)	0.81020 (19)	0.93959 (8)	0.0478 (5)	
C2	0.1042 (3)	0.7639 (2)	0.98102 (10)	0.0648 (7)	
H2	0.0469	0.8116	1.0057	0.078*	
C3	0.0903 (3)	0.6468 (2)	0.98559 (11)	0.0714 (7)	
H3	0.0236	0.6159	1.0137	0.086*	
C4	0.1735 (3)	0.5744 (2)	0.94915 (11)	0.0706 (7)	
H4	0.1627	0.4954	0.9524	0.085*	
C5	0.2728 (3)	0.62049 (19)	0.90787 (11)	0.0578 (6)	
H5	0.3295	0.5720	0.8834	0.069*	
C6	0.2897 (2)	0.73866 (18)	0.90213 (8)	0.0443 (5)	
C7	0.3947 (2)	0.78523 (18)	0.85793 (9)	0.0446 (5)	
H7	0.4513	0.7348	0.8345	0.054*	
C8	0.5154 (2)	0.93012 (18)	0.80204 (8)	0.0425 (5)	
H8	0.5719	0.8644	0.7865	0.051*	
C9	0.4216 (2)	0.98194 (16)	0.75096 (8)	0.0413 (4)	
C10	0.3234 (2)	1.06841 (19)	0.76429 (10)	0.0518 (5)	
H10	0.3131	1.0932	0.8043	0.062*	
C11	0.2389 (3)	1.1196 (2)	0.71834 (12)	0.0573 (6)	
H11	0.1733	1.1779	0.7294	0.069*	
C12	0.3404 (2)	1.00365 (19)	0.64556 (10)	0.0494 (5)	
C13	0.3487 (3)	0.9714 (2)	0.58382 (10)	0.0596 (6)	
H13	0.2916	1.0107	0.5552	0.071*	
C14	0.4382 (3)	0.8840 (2)	0.56510 (9)	0.0601 (6)	
H14	0.4423	0.8643	0.5241	0.072*	
C15	0.5242 (2)	0.82375 (19)	0.60786 (9)	0.0495 (5)	
C16	0.5222 (2)	0.85392 (18)	0.66807 (9)	0.0458 (5)	
H16	0.5816	0.8140	0.6957	0.055*	
C17	0.4310 (2)	0.94490 (17)	0.68904 (8)	0.0410 (4)	
C18	0.6243 (2)	1.02128 (19)	0.82638 (8)	0.0465 (5)	
H18	0.5647	1.0885	0.8377	0.056*	
C19	0.7364 (3)	1.0596 (2)	0.77607 (10)	0.0639 (7)	
H19A	0.7236	1.0129	0.7399	0.077*	
H19B	0.7184	1.1390	0.7652	0.077*	
C20	0.8952 (3)	1.0458 (2)	0.80107 (11)	0.0639 (6)	
H20	0.9683	1.0689	0.7701	0.077*	
C21	0.9177 (3)	0.9201 (2)	0.81741 (14)	0.0804 (8)	
H21A	1.0195	0.9076	0.8313	0.096*	
H21B	0.9003	0.8724	0.7819	0.096*	
C22	0.8070 (3)	0.8886 (2)	0.86801 (12)	0.0674 (7)	
H22A	0.7502	0.8216	0.8558	0.081*	
H22B	0.8621	0.8693	0.9047	0.081*	
C23	0.7916 (3)	1.0797 (2)	0.90419 (10)	0.0636 (6)	
H23A	0.8376	1.0582	0.9426	0.076*	
H23B	0.7251	1.1438	0.9119	0.076*	
C24	0.9161 (3)	1.1181 (2)	0.85889 (11)	0.0603 (6)	
H24	1.0135	1.0987	0.8768	0.072*	
C25	0.9125 (3)	1.2447 (2)	0.84784 (13)	0.0727 (7)	
H25	0.8233	1.2750	0.8330	0.087*	
C26	1.0210 (4)	1.3160 (3)	0.85690 (14)	0.0932 (9)	
H26A	1.1125	1.2899	0.8717	0.112*	
H26B	1.0077	1.3935	0.8486	0.112*	
C27	0.6104 (4)	0.6886 (3)	0.53269 (10)	0.0893 (9)	
H27A	0.6449	0.7465	0.5050	0.134*	
H27B	0.5099	0.6670	0.5222	0.134*	
H27C	0.6746	0.6228	0.5300	0.134*	

### Atomic displacement parameters (Å^2^)

**Table:**

	*U*^11^	*U*^22^	*U*^33^	*U*^12^	*U*^13^	*U*^23^
N1	0.0486 (9)	0.0457 (10)	0.0395 (9)	−0.0028 (8)	0.0046 (7)	0.0016 (7)
N2	0.0560 (11)	0.0516 (11)	0.0712 (13)	0.0021 (10)	−0.0051 (10)	0.0136 (10)
N3	0.0598 (11)	0.0577 (11)	0.0426 (9)	−0.0051 (10)	−0.0053 (8)	−0.0014 (8)
O1	0.0809 (11)	0.0513 (9)	0.0596 (9)	0.0002 (9)	0.0226 (9)	−0.0035 (8)
O2	0.0864 (11)	0.0694 (11)	0.0469 (8)	0.0073 (10)	0.0076 (8)	−0.0099 (8)
C1	0.0538 (12)	0.0507 (13)	0.0390 (10)	−0.0019 (10)	0.0019 (10)	0.0014 (9)
C2	0.0707 (15)	0.0719 (17)	0.0517 (13)	−0.0034 (14)	0.0177 (12)	0.0014 (12)
C3	0.0781 (17)	0.0786 (18)	0.0574 (14)	−0.0175 (15)	0.0167 (13)	0.0138 (13)
C4	0.0897 (18)	0.0547 (15)	0.0674 (15)	−0.0137 (15)	0.0075 (14)	0.0125 (13)
C5	0.0679 (14)	0.0463 (13)	0.0591 (13)	−0.0017 (12)	0.0074 (12)	0.0038 (11)
C6	0.0482 (11)	0.0473 (12)	0.0375 (9)	−0.0018 (9)	−0.0014 (9)	0.0036 (9)
C7	0.0479 (11)	0.0454 (12)	0.0405 (10)	0.0012 (10)	0.0033 (9)	−0.0003 (9)
C8	0.0442 (10)	0.0453 (11)	0.0379 (9)	−0.0011 (9)	0.0046 (8)	0.0007 (9)
C9	0.0408 (10)	0.0394 (10)	0.0438 (10)	−0.0071 (9)	0.0025 (8)	0.0045 (9)
C10	0.0516 (12)	0.0479 (12)	0.0559 (12)	0.0017 (11)	0.0078 (10)	0.0002 (10)
C11	0.0530 (12)	0.0456 (12)	0.0734 (16)	0.0042 (11)	0.0064 (11)	0.0093 (11)
C12	0.0488 (11)	0.0470 (12)	0.0523 (12)	−0.0088 (10)	−0.0035 (10)	0.0095 (10)
C13	0.0674 (14)	0.0602 (14)	0.0510 (12)	−0.0078 (13)	−0.0152 (11)	0.0141 (11)
C14	0.0752 (15)	0.0676 (16)	0.0375 (11)	−0.0139 (14)	−0.0051 (11)	0.0019 (11)
C15	0.0566 (12)	0.0479 (12)	0.0441 (11)	−0.0086 (11)	0.0055 (10)	−0.0015 (9)
C16	0.0497 (11)	0.0465 (12)	0.0413 (10)	−0.0045 (10)	−0.0020 (9)	0.0030 (9)
C17	0.0401 (10)	0.0406 (11)	0.0422 (9)	−0.0085 (8)	−0.0010 (8)	0.0040 (9)
C18	0.0502 (11)	0.0476 (12)	0.0415 (10)	−0.0052 (10)	0.0022 (9)	−0.0005 (9)
C19	0.0603 (13)	0.0809 (17)	0.0504 (12)	−0.0277 (14)	0.0008 (11)	0.0005 (13)
C20	0.0519 (12)	0.0726 (17)	0.0672 (14)	−0.0130 (12)	0.0106 (11)	−0.0142 (13)
C21	0.0683 (15)	0.0689 (18)	0.104 (2)	−0.0005 (14)	0.0052 (16)	−0.0314 (16)
C22	0.0717 (15)	0.0593 (15)	0.0713 (15)	0.0022 (13)	−0.0193 (13)	−0.0007 (12)
C23	0.0668 (14)	0.0665 (15)	0.0574 (13)	−0.0067 (13)	−0.0074 (12)	−0.0147 (12)
C24	0.0481 (12)	0.0574 (14)	0.0755 (15)	−0.0019 (11)	−0.0123 (11)	−0.0088 (12)
C25	0.0646 (15)	0.0635 (16)	0.0900 (19)	0.0015 (14)	−0.0104 (14)	−0.0072 (14)
C26	0.091 (2)	0.0693 (19)	0.119 (2)	−0.0187 (18)	−0.003 (2)	−0.0112 (18)
C27	0.133 (3)	0.084 (2)	0.0506 (13)	0.006 (2)	0.0223 (16)	−0.0132 (14)

### Geometric parameters (Å, °)

**Table:**

N1—C7	1.269 (3)	C13—C14	1.359 (3)
N1—C8	1.468 (2)	C13—H13	0.9300
N2—C11	1.317 (3)	C14—C15	1.402 (3)
N2—C12	1.367 (3)	C14—H14	0.9300
N3—C18	1.466 (3)	C15—C16	1.367 (3)
N3—C22	1.468 (3)	C16—C17	1.415 (3)
N3—C23	1.468 (3)	C16—H16	0.9300
O1—C1	1.361 (3)	C18—C19	1.556 (3)
O1—H1	0.8200	C18—H18	0.9800
O2—C15	1.369 (3)	C19—C20	1.529 (3)
O2—C27	1.422 (3)	C19—H19A	0.9700
C1—C2	1.380 (3)	C19—H19B	0.9700
C1—C6	1.401 (3)	C20—C21	1.525 (4)
C2—C3	1.377 (4)	C20—C24	1.535 (3)
C2—H2	0.9300	C20—H20	0.9800
C3—C4	1.381 (4)	C21—C22	1.531 (4)
C3—H3	0.9300	C21—H21A	0.9700
C4—C5	1.377 (3)	C21—H21B	0.9700
C4—H4	0.9300	C22—H22A	0.9700
C5—C6	1.394 (3)	C22—H22B	0.9700
C5—H5	0.9300	C23—C24	1.558 (3)
C6—C7	1.455 (3)	C23—H23A	0.9700
C7—H7	0.9300	C23—H23B	0.9700
C8—C9	1.524 (3)	C24—C25	1.498 (4)
C8—C18	1.537 (3)	C24—H24	0.9800
C8—H8	0.9800	C25—C26	1.293 (4)
C9—C10	1.369 (3)	C25—H25	0.9300
C9—C17	1.428 (3)	C26—H26A	0.9300
C10—C11	1.394 (3)	C26—H26B	0.9300
C10—H10	0.9300	C27—H27A	0.9600
C11—H11	0.9300	C27—H27B	0.9600
C12—C13	1.408 (3)	C27—H27C	0.9600
C12—C17	1.426 (3)		
			
C7—N1—C8	118.14 (18)	C16—C17—C12	118.01 (18)
C11—N2—C12	116.44 (19)	C16—C17—C9	124.77 (17)
C18—N3—C22	111.69 (17)	C12—C17—C9	117.22 (18)
C18—N3—C23	107.62 (18)	N3—C18—C8	112.13 (17)
C22—N3—C23	107.80 (17)	N3—C18—C19	111.27 (17)
C1—O1—H1	109.5	C8—C18—C19	111.09 (16)
C15—O2—C27	119.3 (2)	N3—C18—H18	107.4
O1—C1—C2	118.6 (2)	C8—C18—H18	107.4
O1—C1—C6	121.00 (18)	C19—C18—H18	107.4
C2—C1—C6	120.4 (2)	C20—C19—C18	108.15 (19)
C3—C2—C1	119.6 (2)	C20—C19—H19A	110.1
C3—C2—H2	120.2	C18—C19—H19A	110.1
C1—C2—H2	120.2	C20—C19—H19B	110.1
C2—C3—C4	121.1 (2)	C18—C19—H19B	110.1
C2—C3—H3	119.4	H19A—C19—H19B	108.4
C4—C3—H3	119.4	C21—C20—C19	107.9 (2)
C5—C4—C3	119.2 (2)	C21—C20—C24	108.6 (2)
C5—C4—H4	120.4	C19—C20—C24	110.6 (2)
C3—C4—H4	120.4	C21—C20—H20	109.9
C4—C5—C6	121.1 (2)	C19—C20—H20	109.9
C4—C5—H5	119.5	C24—C20—H20	109.9
C6—C5—H5	119.5	C20—C21—C22	108.5 (2)
C5—C6—C1	118.5 (2)	C20—C21—H21A	110.0
C5—C6—C7	120.0 (2)	C22—C21—H21A	110.0
C1—C6—C7	121.46 (19)	C20—C21—H21B	110.0
N1—C7—C6	122.49 (19)	C22—C21—H21B	110.0
N1—C7—H7	118.8	H21A—C21—H21B	108.4
C6—C7—H7	118.8	N3—C22—C21	111.8 (2)
N1—C8—C9	107.63 (15)	N3—C22—H22A	109.2
N1—C8—C18	110.92 (15)	C21—C22—H22A	109.2
C9—C8—C18	109.15 (16)	N3—C22—H22B	109.2
N1—C8—H8	109.7	C21—C22—H22B	109.2
C9—C8—H8	109.7	H22A—C22—H22B	107.9
C18—C8—H8	109.7	N3—C23—C24	112.82 (18)
C10—C9—C17	117.65 (18)	N3—C23—H23A	109.0
C10—C9—C8	119.18 (18)	C24—C23—H23A	109.0
C17—C9—C8	123.17 (17)	N3—C23—H23B	109.0
C9—C10—C11	120.6 (2)	C24—C23—H23B	109.0
C9—C10—H10	119.7	H23A—C23—H23B	107.8
C11—C10—H10	119.7	C25—C24—C20	114.0 (2)
N2—C11—C10	124.5 (2)	C25—C24—C23	111.8 (2)
N2—C11—H11	117.7	C20—C24—C23	106.36 (18)
C10—C11—H11	117.7	C25—C24—H24	108.2
N2—C12—C13	117.4 (2)	C20—C24—H24	108.2
N2—C12—C17	123.57 (19)	C23—C24—H24	108.2
C13—C12—C17	119.0 (2)	C26—C25—C24	126.5 (3)
C14—C13—C12	121.5 (2)	C26—C25—H25	116.8
C14—C13—H13	119.2	C24—C25—H25	116.8
C12—C13—H13	119.2	C25—C26—H26A	120.0
C13—C14—C15	119.8 (2)	C25—C26—H26B	120.0
C13—C14—H14	120.1	H26A—C26—H26B	120.0
C15—C14—H14	120.1	O2—C27—H27A	109.5
C16—C15—O2	115.9 (2)	O2—C27—H27B	109.5
C16—C15—C14	120.6 (2)	H27A—C27—H27B	109.5
O2—C15—C14	123.51 (18)	O2—C27—H27C	109.5
C15—C16—C17	121.0 (2)	H27A—C27—H27C	109.5
C15—C16—H16	119.5	H27B—C27—H27C	109.5
C17—C16—H16	119.5		
			
O1—C1—C2—C3	−179.6 (2)	C15—C16—C17—C9	179.84 (19)
C6—C1—C2—C3	0.2 (4)	N2—C12—C17—C16	178.52 (19)
C1—C2—C3—C4	−0.4 (4)	C13—C12—C17—C16	−1.9 (3)
C2—C3—C4—C5	0.5 (4)	N2—C12—C17—C9	−0.9 (3)
C3—C4—C5—C6	−0.4 (4)	C13—C12—C17—C9	178.69 (19)
C4—C5—C6—C1	0.2 (3)	C10—C9—C17—C16	−177.42 (18)
C4—C5—C6—C7	−179.6 (2)	C8—C9—C17—C16	2.7 (3)
O1—C1—C6—C5	179.7 (2)	C10—C9—C17—C12	1.9 (3)
C2—C1—C6—C5	−0.2 (3)	C8—C9—C17—C12	−177.90 (17)
O1—C1—C6—C7	−0.5 (3)	C22—N3—C18—C8	67.6 (2)
C2—C1—C6—C7	179.7 (2)	C23—N3—C18—C8	−174.22 (17)
C8—N1—C7—C6	−176.87 (16)	C22—N3—C18—C19	−57.5 (2)
C5—C6—C7—N1	178.6 (2)	C23—N3—C18—C19	60.7 (2)
C1—C6—C7—N1	−1.3 (3)	N1—C8—C18—N3	53.4 (2)
C7—N1—C8—C9	109.5 (2)	C9—C8—C18—N3	171.84 (16)
C7—N1—C8—C18	−131.2 (2)	N1—C8—C18—C19	178.63 (18)
N1—C8—C9—C10	54.8 (2)	C9—C8—C18—C19	−63.0 (2)
C18—C8—C9—C10	−65.7 (2)	N3—C18—C19—C20	−0.7 (3)
N1—C8—C9—C17	−125.40 (19)	C8—C18—C19—C20	−126.4 (2)
C18—C8—C9—C17	114.15 (19)	C18—C19—C20—C21	59.8 (3)
C17—C9—C10—C11	−1.5 (3)	C18—C19—C20—C24	−58.9 (3)
C8—C9—C10—C11	178.35 (19)	C19—C20—C21—C22	−62.9 (3)
C12—N2—C11—C10	1.3 (3)	C24—C20—C21—C22	57.0 (3)
C9—C10—C11—N2	−0.2 (3)	C18—N3—C22—C21	54.8 (3)
C11—N2—C12—C13	179.7 (2)	C23—N3—C22—C21	−63.2 (2)
C11—N2—C12—C17	−0.7 (3)	C20—C21—C22—N3	5.9 (3)
N2—C12—C13—C14	−178.8 (2)	C18—N3—C23—C24	−63.8 (2)
C17—C12—C13—C14	1.6 (3)	C22—N3—C23—C24	56.8 (3)
C12—C13—C14—C15	0.3 (3)	C21—C20—C24—C25	174.1 (2)
C27—O2—C15—C16	174.0 (2)	C19—C20—C24—C25	−67.7 (3)
C27—O2—C15—C14	−5.4 (3)	C21—C20—C24—C23	−62.3 (3)
C13—C14—C15—C16	−1.8 (3)	C19—C20—C24—C23	56.0 (3)
C13—C14—C15—O2	177.6 (2)	N3—C23—C24—C25	129.9 (2)
O2—C15—C16—C17	−178.10 (18)	N3—C23—C24—C20	4.9 (3)
C14—C15—C16—C17	1.4 (3)	C20—C24—C25—C26	−116.8 (3)
C15—C16—C17—C12	0.5 (3)	C23—C24—C25—C26	122.6 (3)

### Hydrogen-bond geometry (Å, °)

**Table:**

*D*—H···*A*	*D*—H	H···*A*	*D*···*A*	*D*—H···*A*
O1—H1···N1	0.82	1.88	2.605 (2)	148.

## References

[bb1] Bruker (2005). *SADABS* Bruker AXS Inc., Madison, Wisconsin, USA.

[bb2] Bruker (2008). *APEX2* and *SAINT* Bruker AXS Inc., Madison, Wisconsin, USA.

[bb20] Brunner, H., Biigler, J. & Nuber, B. (1995). *Tetrahedron Asymmetry*, **6**, 1699–1702.

[bb21] He, W., Liu, P., Zhang, B.-L. Sun, X.-L. & Zhang, S.-Y. (2006). *Appl. Organometal. Chem.* **20**, 328–334.

[bb3] Hooft, R. W. W., Straver, L. H. & Spek, A. L. (2008). *J. Appl. Cryst.* **41**, 96–103. 10.1107/S0021889807059870 PMC2467520 19461838

[bb4] Jennings, W. B. & Lovely, C. J. (1991). *Tetrahedron*, **41**, 5561–5568.

[bb5] Jones, P. G. (1986). *Acta Cryst.* A**42**, 57.

[bb6] Macrae, C. F., Edgington, P. R., McCabe, P., Pidcock, E., Shields, G. P., Taylor, R., Towler, M. & van de Streek, J. (2006). *J. Appl. Cryst.* **39**, 453–457.

[bb7] Sheldrick, G. M. (2008). *Acta Cryst.* A**64**, 112–122. 10.1107/S0108767307043930 18156677

[bb8] Xie, Y.-S., Dong, W.-L., He, L.-P., Zhang, X.-L. & Zhao, B.-X. (2010). *Acta Cryst.* E**66**, o3106. 10.1107/S160053681004448X PMC3011393 21589412

[bb9] Yin, H. D., Wang, Q. B. & Xue, S. C. (2004). *J. Organomet. Chem.* **689**, 2480–2485.

[bb10] Yoon, T. P. & Jacobsen, E. N. (2005). *Angew. Chem. Int. Ed.* **44**, 466–468. 10.1002/anie.200461814 15624148

[bb11] Zhu, H.-Y. (2011). *Acta Cryst.* E**67**, o812. 10.1107/S1600536811008014 PMC3099914 21754098

